# World assumptions, posttraumatic stress and quality of life after a natural disaster: A longitudinal study

**DOI:** 10.1186/1477-7525-10-76

**Published:** 2012-06-28

**Authors:** Egil Nygaard, Trond Heir

**Affiliations:** 1 Norwegian Centre for Violence and Traumatic Stress Studies, Kirkeveien 166, Bygning 48, 0450, Oslo, Norway

**Keywords:** Natural disaster, Posttraumatic stress, PTSD, Quality of life, World assumptions

## Abstract

**Background:**

Changes in world assumptions are a fundamental concept within theories that explain posttraumatic stress disorder. The objective of the present study was to gain a greater understanding of how changes in world assumptions are related to quality of life and posttraumatic stress symptoms after a natural disaster.

**Methods:**

A longitudinal study of 574 Norwegian adults who survived the Southeast Asian tsunami in 2004 was undertaken. Multilevel analyses were used to identify which factors at six months post-tsunami predicted quality of life and posttraumatic stress symptoms two years post-tsunami.

**Results:**

Good quality of life and posttraumatic stress symptoms were negatively related. However, major differences in the predictors of these outcomes were found. Females reported significantly higher quality of life and more posttraumatic stress than men. The association between level of exposure to the tsunami and quality of life seemed to be mediated by posttraumatic stress. Negative perceived changes in the assumption “the world is just” were related to adverse outcome in both quality of life and posttraumatic stress. Positive perceived changes in the assumptions “life is meaningful” and “feeling that I am a valuable human” were associated with higher levels of quality of life but not with posttraumatic stress.

**Conclusions:**

Quality of life and posttraumatic stress symptoms demonstrate differences in their etiology. World assumptions may be less specifically related to posttraumatic stress than has been postulated in some cognitive theories.

## Background

Natural disasters have a negative impact on individuals’ mental health. Not only do disaster survivors have an increased risk of developing posttraumatic stress disorder (PTSD) [1] and other mental ailments [2], but their quality of life may also be curtailed [3-5]. Factors that increase the risk of developing PTSD include being female [6] and the severity and proximity to the disaster [1]. Social support has been identified as a protective factor [7,8]. Findings concerning the effect of age have been inconsistent [1]. Quality of life and PTSD are highly negatively related in the aftermath of natural disasters [5,9,10]. However, it is unknown whether there are different risk factors for PTSD and decreased quality of life after natural disasters. Findings from the few studies conducted following other traumatic experiences, such as females’ experience with breast cancer [11] and children’s experience with traffic injuries [12], indicate that the risk and protective factors may be quite similar. However, to our knowledge, no study has compared risk factors for PTSD and decreased quality of life after natural disasters. In addition, there is a lack of investigations on changes in world assumptions after a disaster. Such changes are a fundamental concept within theories that explain PTSD.

Several theories have been presented to explain the development of and recovery from PTSD. Cognitive-oriented theories are most commonly referenced in the clinical literature and are the most fully developed and studied e.g., [13]. Ronnie Janoff-Bulman [14-16] has been a major theoretical contributor to the area of PTSD. She concentrated on the nature of trauma victims’ pre-existing assumptions about the world and themselves that she predicted would be shattered during a traumatic event. Similar terms and concepts have been central in cognitive theories for a long time, e.g. “assumptive world” by Parkes [17], “working models” by Bowlby [18], “self-theory/world-theory” by Epstein [19] and Beck's “Cognitive Triad” with negative thoughts about the self, the world, and the future“[20]. Thus, Janoff-Bulman’s theories of world assumptions have a solid theoretical background. The shattering of world assumptions remains a central perspective in cognitive theories of mental health following traumatic experiences e.g., [21].

Many studies have found that people with posttraumatic stress symptoms have more negative world assumptions than people without such reactions e.g., [22-24]. For example, low self-worth has been strongly related to high levels of posttraumatic stress symptoms across different types of traumatic experiences e.g., [22,23,25-27]. Low levels of assumption of benevolence of the world or other people have been related to greater posttraumatic stress symptoms e.g., [22-26,28]. Assumptions such as meaningfulness, justice, luck, randomness, controllability, and self-control have, to a lesser degree, been related to posttraumatic stress symptoms e.g., [25-30].

According to Janoff-Bulman [14], the assumption that is most influenced by natural disasters is that of the meaningfulness of the world, followed by the assumptions of randomness and controllability. In contrast, benevolence of the world, benevolence of persons, and self-worth are more vulnerable after human-induced victimization. However, the few small studies conducted after natural disasters found that self-worth and benevolence of the world were related to the levels of posttraumatic stress symptoms [31-33].

Most studies have investigated which world assumptions people have rather than changes in their assumptions. In one study, participants who had experienced a shipping disaster were retrospectively asked about change in world assumptions following the disaster. They reported strong positive changes in their outlook on life [34]. However, the only two studies we found that investigated world assumptions over time found few [30] or no [27] changes in world assumptions from a few days after trauma until more than six months post-disaster. It is therefore difficult to determine whether world assumptions change after traumatic experiences.

The temporal relations among world assumptions, posttraumatic stress symptoms, and other mental health issues remain unresolved. Two studies that asked participants about change in world assumptions reported that negative changes were related to higher levels of posttraumatic stress symptoms, whereas positive changes in world assumptions were not related to the level of distress [34,35]. We found only six longitudinal studies that investigated the relation between world assumptions and PTSD [26-28,30,35,36], none of which investigated samples who had experienced a natural disaster. Most of the few studies that investigated world assumptions in relation to both posttraumatic stress symptoms and other mental health aspects found that similar world assumptions were related to posttraumatic stress symptoms and other measures of mental health, such as depression, anxiety, grief, quality of life, and general mental health [23,25,27,34,35,37]. However, none of these studies found that world assumptions were more highly related to posttraumatic stress symptoms than to assessments of other mental health issues. Thus, there may not be a specific causal relation between world assumptions and posttraumatic stress symptoms, as suggested by Janoff-Bulman [14] and others [21,38]; instead, the relations may be similar to, and a reflection of, a general connection between world assumptions and mental health.

### Present study: Context and aims

The tsunami in Southeast Asia on December 26, 2004 was the deadliest tsunami in recorded history, with a death toll of approximately 230,000 people [39]. An estimated 4,000 Norwegian citizens were in the affected areas during the tsunami, most of whom were tourists on Christmas vacation. The Norwegian death toll included 58 adults and 26 children. The surviving Norwegians were evacuated to their homes and communities soon after the disaster. This situation thereby provided a unique vantage point for assessing the effects of trauma after a disaster when the post-disaster environment includes minimal secondary stressors.

After the tsunami, the Norwegian authorities initiated a research program that included the present study. The main aim of the present study was to gain a greater understanding of the etiology of posttraumatic stress symptoms and quality of life after natural disasters. Three research questions were investigated. First, were quality of life and posttraumatic stress symptoms negatively related over time? Second, what perceived changes in world assumptions were reported in the aftermath of the disaster? Third, were perceived changes in world assumptions similarly related to later quality of life and posttraumatic stress symptoms?

## Methods

### Procedure

Shortly after the 2004 tsunami in Southeast Asia, the Norwegians who were evacuated from the disaster-stricken areas were registered upon arrival in Norway. A postal questionnaire was sent to all registered persons 18 years or older (*N* = 2,468) at six months (T_1_) and two years (T_2_) post-tsunami. The questionnaire at T_1_ included questions concerning level of exposure, world assumptions, quality of life, posttraumatic stress symptoms, and background information [40]. The questionnaire at T_2_ included questions about quality of life and posttraumatic stress symptoms [41]. The study was approved by the Norwegian Social Science Data Services and the Regional Committee for Medical Research Ethics.

### Participants

A total of 868 and 1,170 subjects responded at T_1_ and T_2_, respectively. We received questionnaires for both T_1_ and T_2_ from 657 respondents. The final analyzed sample included 574 respondents after the exclusion of 83 respondents due to missing data. At T_1_, 63.9% of the participants had more than 12 years of education, and 75.6% were employed. There were multiple participants from the same household in 181 cases (31.5%). More descriptive information about the participants is presented in Table 1.

**Table 1:** Descriptive statistics of the major study variables (N = 574)

**Variable**	**n (%)/M (SD)**
Number of participants from household	
One	393 (68.5%)
Two	156 (27.2%)
Three	21 (3.7%)
Four	4 (0.7%)
Sex	
Male	261 (45.5%)
Female	313 (54.5%)
Age at time of tsunami, years, mean (*SD*)	42.6 (12.7)
Exposure	
Not exposed	105 (18.3%)
Exposed, but no danger	262 (45.6%)
In danger	207 (36.1%)

Note: Demographic variables and exposure were assessed six months post-tsunami.

Non-responders at T_1_ were more likely than responders to have been exposed to less severely affected locations in Southeast Asia [40] and were more often men; however, they were similar in age to responders [42]. The most frequently reported reasons for not participating in the study were lack of interest or time, followed by lack of relevant experiences [43]. The final sample did not differ from responders who were excluded from the analyses on most background features (number of participants from the same household, employment, age, or exposure), quality of life, posttraumatic stress symptoms at T_1_, or most world assumptions.

### Measures

#### Exposure and immediate response to the disaster

Based on earlier work [44], questions regarding a broad spectrum of potential experiences during a tsunami were included in the questionnaire six months post-tsunami. Based on earlier evaluations of the exposure experiences as risk factors [40], the participants were classified into three groups of exposure severity. The “danger-exposed group” consisted of individuals who were directly exposed to the waves (and were caught or almost caught by the waves). The “non-danger-exposed group” included participants who experienced the tsunami but were not in an immediate life-threatening situation (was physically injured without direct contact with the waves, a close relative was injured, experienced fear for the safety of a relative, or witnessed others’ deaths and/or suffering). The “non-exposed group” consisted of participants who were present in Southeast Asia at the time of the tsunami but without the abovementioned exposure to the tsunami [45].

#### World assumptions

The participants were asked to rate the degree to which their following world assumptions were perceived to have changed post-tsunami: the feeling that I will always fare well; the world is just; the world is predictable; the world is controllable; the world is good and benevolent; life is meaningful; and the feeling that I am a valuable human. All seven statements had five response alternatives (*very weakened* = −2, *some weakening* = −1, *unchanged* = 0, *some strengthening* = 1, *very strengthened* = 2). The statements were developed for the present study to capture important features of the World Assumptions Scale [16].

#### Quality of life

Cantril’s vertical visual-analog scale Ladder of Life Satisfaction was used to measure quality of life at both assessment times [46]. The participants were asked to indicate which step on a 10-step ladder currently represented their life. The bottom step represented the *worst life* (1) the participant could imagine, whereas the top step represented the *best life* (10) imaginable. As measures of global quality of life, visual-analog scales have good validity and reliability [47]. At six months post-tsunami, the participants also retrospectively reported their location on the ladder one year before T_1_.

#### Posttraumatic stress symptoms

The Impact of Event Scale-Revised (IES-R) [48] was included at both assessments to measure the participants’ level of posttraumatic stress symptoms during the previous two weeks. The IES-R consists of 22 items with five response alternatives of degree of distress (0 = *not at all*, 1 = *a little*, 2 = *moderately*, 3 = *quite a bit*, 4 = *extremely*). Total mean scores were based on all items. The psychometric properties of the IES-R have been extensively evaluated and deemed acceptable [49]. The IES-R has also been found to have acceptable reliability in a Norwegian non-clinical sample [50]. The internal consistency of this measure was high in the present sample (Cronbach’s α = .95 and .96 at T_1_ and T_2,_ respectively).

### Data analysis

Participants who were missing more than four replies to questions about posttraumatic stress symptoms were excluded. For the remaining participants, missing values for posttraumatic stress symptoms were replaced using expectation maximization algorithms (EM algorithms) [51]. The procedures for handling of missing data were decided in advance according to previous procedures [52]. Dropout analyses were conducted using *χ*^2^-tests for categorical data and student t-tests for continuous data. Student t-tests and Pearson correlations were used to analyze the relation between quality of life and posttraumatic stress symptoms over time. One-sample *t*-test was used to analyze whether the reported perceived change in world assumptions was significantly different from the value of no perceived change (expected mean = 0).

There were significant similarities in mental health between individuals in the same household (intra-class correlations = .40 and .46 for quality of life and posttraumatic stress symptoms, respectively); thus, a multilevel approach was used. Mixed effects models were used to test whether quality of life and posttraumatic stress symptoms at T_2_ could be predicted from information at T_1_. In addition to bivariate analyses, three mixed effects models were tested for both of the dependent variables. A model without predictors (not shown) was used as the base for calculating how much variance was explained by the inclusion of the predictors. Model A included gender, age, exposure, and world assumptions as predictors. Model B included the Model A predictors and either posttraumatic stress symptoms as a predictor of later quality of life or quality of life as a predictor of later posttraumatic stress symptoms. All models controlled for participants who lived in the same household. Due to this multilevel approach, the regression model has error terms at two levels, the individual level and the household level.

All tests were two-tailed, with a significance level of *p* ≤ .05. Statistical analyses were performed using PASW Statistics, version 18.

## Results

The participants reported, retrospectively, that their quality of life was better prior to the tsunami (M = 7.6, SD =1.6) than at T_1_ (t(572) = 4.12, p < .001) or T_2_ (t(572) = 3.13, p = .002). The quality of life and posttraumatic stress symptom reports at T_1_ and T_2_ are presented in Table 2. Quality of life did not change significantly from T_1_ to T_2_. There was a significant, although small, decrease in level of posttraumatic stress symptoms from T_1_ to T_2_. Concurrent quality of life and posttraumatic stress symptoms were highly related at both T_1_ and T_2_.

**Table 2:** Quality of life and posttraumatic stress symptoms at six months and two years post-disaster (N = 574)

	**Six months**	**Two years**	**Change**	**Correlation T**_1_*T_**2**_
	**M (SD)**	**M (SD)**	**M (SD)**	
Quality of life^a^	7.2 (1.9)	7.3 (1.8)	0.1 (1.6)	0.63***
Posttraumatic stress^b^	1.1 (0.8)	1.0 (0.8)	−0.1 (0.6)***	0.76***
Correlation	-0.43***	-0.46***	0.22***	

Note: Pearson’s r was used for correlations, whereas the difference over time was analyzed with Student’s *t*-test.

^a^ Ladder of Life Satisfaction.

^b^ Impact of Event Scale-Revised.

* *p* ≤ .05; ** *p* ≤ .01; *** *p* ≤ .001.

At T_1,_ the participants retrospectively reported a weakening in the feeling that they will always fare well. They also reported a weakening in the four assumptions about the world being just, predictable, controllable, and benevolent in the aftermath of the tsunami (each p < .001). On the other hand, the participants reported a strengthening of their assumptions of life as meaningful and being a valuable human (each p < .001) (Table 3).

**Table 3:** Retrospective evaluation of perceived changes in world assumptions at six months post-tsunami (N = 574)

**Variable**	**M (SD)**
Invulnerability	−0.2 (0.9)
Just world	−0.6 (0.9)
Predictable world	−0.8 (1.0)
Controllable world	−0.8 (0.9)
Good and benevolent world	−0.5 (0.9)
Meaningful life	0.4 (1.0)
Valuable person	0.3 (0.8)

Note: All means are significantly different from zero (*p* ≤ .001).

Bivariate and multivariate regression analyses for the prospective prediction of quality of life and posttraumatic stress symptoms are presented in Tables 4 and 5, respectively. In general, independent variables that were positively related to better quality of life were negatively related to posttraumatic stress symptoms. Exceptions were found for gender and age. Females had both better quality of life and more posttraumatic stress symptoms than males, both in the bivariate analyses (Table 4) and in the analyses that adjusted for other variables (Table 5). Age was not significantly related to quality of life or posttraumatic stress symptoms in the bivariate analyses but was significantly positively associated with quality of life when other variables were taken into account.

**Table 4:** Bivariate mixed effects analyses predicting quality of life and posttraumatic stress symptoms two years post-tsunami (N = 574)

**Variable**	**Quality of life at T**_**2**_	**Posttraumatic stress at T**_**2**_
	**b (95% CI)**	**b (95% CI)**
Gender		
Male	−0.25 (−0.40, -0.10)***	−0.27 (−0.41, -0.12)***
Female^a^	0 (0)	0 (0)
Age	.00 (−0.00, 0.01)	.00 (−0.01, 0.01)
Exposure		
Not exposed	0.29 (0.05, 0.53)*	−1.01 (−1.24, -0.79)***
Exposed, but no danger	0.22 (0.04, 0.41)*	−0.48 (−0.67, -0.31)***
In danger^a^	0 (0)	0 (0)
Invulnerability	0.11 (0.03, 0.20)**	−0.17 (−0.26, -0.08)***
Just world	0.22 (0.12, 0.31)***	−0.29 (−0.38, -0.20)***
Predictable world	0.06 -(0.01, 0.14)	−0.11 (−0.19, -0.03)**
Controllable world	0.10 (0.02, 0.19)*	−0.24 (−0.33, -0.15)***
Good and benevolent world	0.22 (0.12, 0.31)***	−0.27 (−0.36, -0.18)***
Meaningful life	0.31 (0.23, 0.39)***	−0.09 (−0.17, -0.00)*
Valuable person	0.37 (0.27, 0.47)***	−0.04 (−0.12, 0.04)
Quality of life at T_1_	0.62 (0.55, 0.68)***	−0.40 (−0.47, 0.32)***
Posttraumatic stress T_1_	−0.34 (−0.42, -0.27)***	0.75 (0.70, 0.81)***

Note: The multilevel regression analysis was controlled for the effect of mutual address. Figures are regression coefficients (confidence intervals in parenthesis). Quality of life and posttraumatic stress symptoms were standardized (Z-values). for comparability. All predictors were measured six months post-tsunami.

^a^ Females and those respondents exposed to danger were set to have a mean of 0 in the mixed effect models.

* *p* ≤ .05; ** *p* ≤ .01; *** *p* ≤ .001.

**Table 5:** Multivariate mixed effects analyses predicting quality of life and posttraumatic stress symptoms two years post-tsunami (N = 574)

	**Model A**		**Model B**	
	**Quality of life**	**Posttraumatic stress**	**Quality of life**	**Posttraumatic stress**
*Fixed effects*				
Intercept	−0.29 (−0.61, 0.22)	0.15 (−0.16, 0.46)	−0.24 (−0.54, 0.06)	0.01 (−0.28, 0.31)
Gender				
Male	−0.25 (−0.40, -0.11)***	−0.20 (−0.35, -0.06)**	−0.36 (−0.50, -0.22)***	−0.22 (−0.35, -0.08)**
Female^a^	0 (0)	0 (0)	0 (0)	0 (0)
Age	0.01 (0.00, 0.01)*	0.00 (−0.00, 0.01)	0.01 (0.00, 0.01)**	0.01 (−0.00, 0.01)
Exposure				
Not exposed	0.27 (0.05, 0.50)*	−0.94 (−1.16, -0.72)***	−0.06 (−0.29, 0.17)	−0.80 (−1.01, -0.59)***
Exposed, but no danger	0.16 (−0.02, 0.33)	−0.41 (−0.58, -0.24)***	0.02 (−0.15, 0.19)	−0.32 (−0.48, -0.16)***
In danger^a^	0 (0)	0 (0)	0 (0)	0 (0)
Invulnerability	0.03 (−0.05, 0.12)	−0.08 (−0.17, 0.00)	−0.02 (−0.10, 0.06)	−0.04 (−0.12, 0.03)
Just world	0.18 (0.08, 0.29)***	−0.17 (−0.27, -0.06)**	0.11 (0.01, 0.22)*	−0.13 (−0.23, -0.03)*
Predictable world	−0.03 (−0.13, 0.06)	0.11 (0.01, 0.20)*	−0.03 (−0.12, 0.07)	0.09 (−0.00, 0.18)
Controllable world	−0.03 (−0.15, 0.09)	−0.09 (−0.21, 0.02)	−0.04 (−0.15, 0.07)	−0.08 (−0.19, 0.03)
Good and benevolent world	0.11 (0.00, 0.22)*	−0.11 (−0.22, -0.01)*	0.07 (−0.03, 0.18)	−0.10 (−0.20, 0.01)
Meaningful life	0.15 (0.05, 0.25)**	−0.08 (−0.18, 0.02)	0.14 (0.05, 0.24)**	−0.01 (−0.11, 0.09)
Valuable person	0.23 (0.11, 0.36)***	−0.01 (−0.13, 0.11)	0.20 (0.09, 0.32)***	0.08 (−0.04, 0.19)
Quality of life at T_1_				−0.35 (−0.42, -0.27)***
Posttraumatic stress at T_1_			−0.34 (−0.42, -0.25)***	
*Explained variance*				
Between households	32.5%	52.7%	44.8%	53.4%
Within households	7.0%	−5.0%	12.3%	11.4%
Total explained variance	17.2%	21.7%	25.3%	30.8%
*Model fit*				
AIC	1,549.66	1,529.64	1,497.33	1,461.54

Note: The multilevel regression analysis was controlled for the effect of mutual address. Figures are regression coefficients (confidence intervals in parenthesis). Quality of life and posttraumatic stress symptoms were standardized (Z-values). All predictors were measured six months post-tsunami. Model A includes all control variables (age, gender, exposure) and world assumptions. Model B also includes quality of life or posttraumatic stress symptoms at six months. The explained variance is the percentage reduction in unexplained variance as compared to a model without any independent variables. The AIC for such an empty model was 1,614.32 and 1,616.05 for quality of life and posttraumatic stress symptoms, respectively.

AIC = Akaike’s information criterion.

^a^ Females and those respondents exposed to danger were set to have a mean of 0 in the mixed effect models.

* *p* ≤ .05; ** *p* ≤ .01; *** *p* ≤ .001.

Whereas non-exposed individuals reported better quality of life than participants who were exposed to danger (Table 4), this effect was not significant after adjusting for posttraumatic stress symptoms (Model B in Table 5). However, exposure was highly related to posttraumatic stress symptoms even after adjusting for quality of life. Thus, the effect of exposure on quality of life was potentially mediated through posttraumatic stress symptoms.

In the final models, a weakening of the world assumption “the world is just” at T_1_ was related to worse quality of life and more posttraumatic stress symptoms at T_2_ (Table 5). Those respondents who reported weakened assumptions about “the world as predictable” had less posttraumatic stress symptoms when controlling for other variables (Model A in Table 5). However, the change from a negative association in the bivariate model to a positive association in the multivariate analyses indicates that this effect may be an artifact from other variables entered into the model. The main difference between how posttraumatic stress symptoms and quality of life were related to world assumptions was found for the assumptions “life is meaningful” and “feeling that I am a valuable human.” Those respondents who reported more strengthening in these two assumptions reported higher quality of life. This result was found both in the bivariate analyses and when controlling for other variables. However, there was no such association between strengthening of personal assumptions and posttraumatic stress symptoms when other variables were controlled.

## Discussion

Although quality of life and posttraumatic stress symptoms were highly related, major differences in predictors were found. Gender, exposure, and world assumptions were related to quality of life in other ways than they were related to posttraumatic stress symptoms.

As reported in many other studies following natural disasters, females reported more posttraumatic stress symptoms than males [6]. However, females also reported better quality of life. This finding is similar to a population-based study in Norway [53], but contrary to studies after earthquakes in Taiwan [3,5,9] and Turkey [54], which found that females reported poorer quality of life than males. The present finding that females had better quality of life than males may be a cultural affinity rather than an effect of the tsunami experiences.

Whereas higher levels of exposure were related to more posttraumatic stress symptoms independently of quality of life, the relationship between level of exposure and quality of life seemed to be mediated through posttraumatic stress symptoms. The participants in the present study were protected against many common secondary adversities in the aftermath of disasters, such as the destruction of homes and disruption of jobs. This protection may explain why exposure was related to worse quality of life only among participants with posttraumatic stress symptoms. This finding is in contrast to studies of victims who live in a disaster-stricken area; these studies found that exposure was a risk factor for decreased quality of life, after controlling for PTSD [3,5] or depression [4].

Perceived changes in world assumptions were marginally related to later posttraumatic stress symptoms but significantly related to later quality of life. There were some differences in associations between world assumptions and the two outcomes. Whereas reported negative perceived changes in the assumption “the world is just” were related to worse outcomes in both quality of life and posttraumatic stress symptoms, only quality of life was related to perceived changes in “life is meaningful” and the “feeling that I am a valuable human.” On average, these two assumptions were reinforced, whereas the five other assumptions were weakened. Thus, positive perceived changes in assumptions about personal attributes seemed to be related to better quality of life but not to posttraumatic stress symptoms. This result is in contrast to several studies that found that self-worth was related to posttraumatic stress symptoms e.g., [22,23,25-27]. This discrepancy may be due to measurement differences because the present study investigated perceived changes in world assumptions whereas the earlier studies measured level of self-worth at the time of the assessment. Thus, the level of self-worth, rather than the change in self-worth, may be related to posttraumatic stress symptoms. The type of trauma may also differentially impact self-worth. For example, human-induced traumas such as terrorist attacks or interpersonal violence may have greater consequences for the assumptions about self-worth [14]. The lack of relationship between posttraumatic stress symptoms and perceived changes in world assumptions was in contrast to Janoff-Bulman’s [14] and other cognitive theories [21,38] of reactions after trauma. The current findings suggest that world assumptions may be more related to mental health in general than to a specific process after traumatic experiences.

However, the results can also be interpreted as the participants experienced posttraumatic growth (positive perceived change in sense of self-worth and meaningfulness of life), with positive consequences for their quality of life. This interpretation would be in accordance with Janoff-Bulman’s claims that posttraumatic growth is an attempt to understand the value or meaning of trauma for one’s life [55]. The world assumptions of trauma survivors may be more realistic post-trauma; at the same time, the survivors may actively concentrate more on some positive aspects of life. A positive reinterpretation of the tsunami experiences may thus buffer the effect of posttraumatic stress symptoms on quality of life [11]. However, a meta-analytic study did not find a relationship between posttraumatic growth and quality of life [56].

As found in previous disaster studies, quality of life and posttraumatic stress symptoms were highly related [5,9,10]. The strength of the relationship between posttraumatic stress symptoms at six months post-tsunami and later quality of life was similar to that of the association between quality of life at six months post-tsunami and later posttraumatic stress symptoms (Table 5). Thus, no indication of the direction of causality between these two measures of mental health was found. There is likely an interactional process through which both aspects of mental health influence each other over time. This process may also include other aspects of mental health, such as depression and anxiety [4,57].

### Methodological considerations

The present study had several methodological advantages and limitations. Nearly all of the Norwegians who were in the afflicted area were invited to participate, thus reducing sample selection bias. The participants experienced a single, easily identifiable trauma and were largely protected against reminders and secondary adversities due to their return to intact homes and rather unaffected communities. Thus, the confounding features that are common in research following natural disasters were reduced.

There was a relatively low response rate. However, due to the directionality of the dropout bias, the included participants seem to represent the most heavily exposed Norwegian tourists in the tsunami-stricken areas [43].

As in most posttraumatic studies, the pre-disaster information was gathered retrospectively or was lacking. Thus, reports of pre-tsunami quality of life may have been influenced by concurrent mental health. Similarly, perceived changes in world assumptions after the tsunami may have been influenced by pre-disaster level of world assumptions and concurrent mental health. The present study investigated perceived changes in world assumptions, rather than absolute levels of world assumptions. Thus, one should be careful when comparing the results with earlier studies of levels of world assumptions.

## Conclusions

Most studies on the relationship between world assumptions and posttraumatic stress symptoms have used the World Assumption Scale [16]. This scale measures concurrent levels of assumptions rather than changes in assumptions. However, theories often indicate how changes in, rather than levels of, world assumptions are related to traumatic experiences. Thus, future studies should include measures of changes in world assumptions.

Quality of life and posttraumatic stress symptoms are highly related. However, there are major differences between the factors that are related to posttraumatic stress symptoms and quality of life after disaster experiences. Thus, studies should have a broader perspective than posttraumatic stress symptoms to understand mental health-related processes after traumatic experiences.

Changes in world assumptions are a fundamental concept within cognitive theories of PTSD e.g., [21]. Findings from the present study indicate that changes in world assumptions are not as specifically related to posttraumatic stress symptoms as suggested by Janoff-Bulman [14,15] but that changes in world assumptions may be even more related to quality of life. Thus, cognitive theories of the importance of changes in world assumptions after traumatic events should in the future also include how changes in world assumptions are related to quality of life.

## Abbreviations

AIC: Akaike’s information criterion; IES-R: Impact of event scale-revised; PTSD: Posttraumatic stress disorder.

## Competing interests

The authors declare that they have no competing interests.

## Authors’ contributions

EN performed the literature review, conducted the statistical analysis and drafted the manuscript. TH conceived the study and its design and contributed to the manuscript. Both authors have read and approved the final manuscript.

## References

[B1] Norris FH Friedman MJ Watson PJ Byrne CM Diaz E Kaniasty K 60,000 disaster victims speak: Part I. An empirical review of the empirical literature, 1981–2001 Psychiatry 2002 65 207 239 10.1521/psyc.65.3.207.20173 12405079

[B2] McFarlane AC Papay P Multiple diagnoses in posttraumatic stress disorder in the victims of a natural disaster J Nerv Ment Dis 1992 180 498 504 10.1097/00005053-199208000-00004 1500931

[B3] Chou FH Chou P Su TT Ou-Yang WC Chien IC Lu MK Huang MW Quality of life and related risk factors in a Taiwanese village population 21 months after an earthquake Aus NZ J Psychiatry 2004 38 358 364 10.1080/j.1440-1614.2004.01364.x 15144515

[B4] Heo JH Kim MH Koh SB Noh S Park JH Ahn JS Park KC Shin J Min S A prospective study on changes in health status following flood disaster Psychiatry Investig 2008 5 186 192 10.4306/pi.2008.5.3.186 PMC2796032 20046364

[B5] Tsai KY Chou P Chou FH Su TT Lin SC Lu MK Ou-Yang WC Su CY Chao SS Huang MW Three-year follow-up study of the relationship between posttraumatic stress symptoms and quality of life among earthquake survivors in Yu-Chi, Taiwan J Psychiatr Res 2007 41 90 96 10.1016/j.jpsychires.2005.10.004 16325854

[B6] Olff M Langeland W Draijer N Gersons BP Gender differences in posttraumatic stress disorder Psychological Bulletin 2007 133 183 204 17338596 10.1037/0033-2909.133.2.183

[B7] Brewin CR Andrews B Valentine JD Meta-analysis of risk factors for posttraumatic stress disorder in trauma-exposed adults J Consult Clin Psychol 2000 68 748 766 11068961 10.1037//0022-006x.68.5.748

[B8] Ozer EJ Best SR Lipsey TL Weiss DS Predictors of posttraumatic stress disorder and symptoms in adults: A meta-analysis Psychological Bulletin 2003 129 52 73 12555794 10.1037/0033-2909.129.1.52

[B9] Chou FH Chou P Lin C Su TT Ou-Yang WC Chien IC Su CY Lui MK Chen MC The relationship between quality of life and psychiatric impairment for a Taiwanese community post-earthquake Quality of Life Research 2004 13 1089 1097 15287275 10.1023/B:QURE.0000031337.73269.64

[B10] Wang X Gao L Shinfuku N Zhang H Zhao C Shen Y Longitudinal study of earthquake-related PTSD in a randomly selected community sample in north China The American Journal of Psychiatry 2000 157 1260 1266 10.1176/appi.ajp.157.8.1260 10910788

[B11] Morill EF Brewer NT O'Neill SC Lillie SE Dees EC Carey LA Rimer BK The interactions of post-traumatic growth and post-traumatic stress symptoms in predicting depressive symptoms and quality of life Psycho-Oncology 2008 17 948 953 10.1002/pon.1313 18213677

[B12] Sturms LM van der Sluis CK Stewart RE Groothoff JW ten Duis HJ Eisma WH A prospective study on paediatric traffic injuries: Health-related quality of life and post-traumatic stress Clin Rehabil 2005 19 312 322 10.1191/0269215505cr867oa 15859532

[B13] Smith P Perrin S Yule W Clark DM Post traumatic stress disorder. Cognitive therapy with children and young people 2010 Routledge, London

[B14] Janoff-Bulman R Shattered assumptions: Towards a new psychology of trauma 1992 Free Press, New York

[B15] Janoff-Bulman R Figley CR The aftermath of victimization: Rebuilding shattered assumptions Trauma and its wake 1985 Brunner/Mazel, New York 15 35

[B16] Janoff-Bulman R Assumptive worlds and the stress of traumatic events: Applications of the schema construct Social Cognition 1989 7 113 136 10.1521/soco.1989.7.2.113

[B17] Parkes CM What becomes of redundant world models? A contribution to the study of adaption to change Bri J Med Psychol 1975 48 131 137 10.1111/j.2044-8341.1975.tb02315.x 1174481

[B18] Bretherton I The origins of attachment theory: John Bowlby and Mary Ainsworth Dev Psychol 1992 28 759 775

[B19] Epstein S The implications of cognitive-experiential self-theory for research in social psychology and personality J Theory Soc Behav 1985 15 283 310 10.1111/j.1468-5914.1985.tb00057.x

[B20] Beck AT Rush AJ Shaw BF Emery G Cognitive therapy of depression 1979 The Guilford Press, New York

[B21] Ehlers A Clark DM A cognitive model of posttramatic stress disorder Behav Res Ther 2000 38 319 345 10.1016/S0005-7967(99)00123-0 10761279

[B22] Elklit A Shevlin M Solomon Z Dekel R Factor structure and concurrent validity of the world assumptions scale J Trauma Stress 2007 20 291 301 10.1002/jts.20203 17598140

[B23] Foa EB Ehlers A Clark DM Tolin DF Orsillo SM The posttraumatic cognitions inventory (PTCI): Development and validation Psychol Assess 1999 11 303 314

[B24] Jind L Do traumatic events influence cognitive schemata? Scand J Psychol 2001 42 113 120 10.1111/1467-9450.00220 11321634

[B25] Solomon S Iancu I Tyano S World assumptions following disaster J Appl Soc Psychol 1997 27 1785 1798 10.1111/j.1559-1816.1997.tb01625.x

[B26] Dekel S Mandl C Solomon Z Shared and unique predictors of post-traumatic growth and distress J Clin Psychol 2011 67 241 252 10.1002/jclp.20747 21254052

[B27] Jeavons S Godber T World Assumptions as a measure of meaning in rural road crash victims Aust J Rural Health 2005 13 226 231 10.1111/j.1440-1584.2005.00706.x 16048464

[B28] Yuan C Wang Z Inslicht SS McCaslin SE Metzler TJ Henn-Haase C Apfel BA Tong H Neylan TC Fang Y Marmar CR Protective factors for posttraumatic stress disorder symptoms in a prospective study of police officers Psychiatry Res 2011 188 45 50 10.1016/j.psychres.2010.10.034 21095622 PMC3071439

[B29] Ullman SE Attributions, world assumptions, and recovery from sexual assault J Child Sex Abuse 1997 6 1 19

[B30] Ginzburg K PTSD and world assumptions following myocardial infarction: A longitudinal study Am J Orthopsychiatry 2004 74 286 292 15291705 10.1037/0002-9432.74.3.286

[B31] Bodvarsdottir I Elklit A Psychological reactions in Icelandic earthquake survivors Scand J Psychol 2004 45 3 13 10.1111/j.1467-9450.2004.00373.x 15016274

[B32] Monson CM Gradus JL La Bash HA Griffin MG Resick PA The role of couples' interacting world assumptions and relationship adjustment in women's postdisaster PTSD symptoms J Trauma Stress 2009 22 276 281 10.1002/jts.20432 19626677 PMC2955403

[B33] Stormyren S Jensen TK Verdensanskuelser etter en katastrofe Tidsskrift for Norsk Psykologforening 2008 12 1498 1506

[B34] Joseph S Williams R Yule W Changes in outlook following disaster: The preliminary development of a measure to assess positive and negative responses J Traumatic Stress 1993 6 271 279 10.1002/jts.2490060209

[B35] Butler LD Koopman C Azarow J Blasey CM Magdalene JC DiMiceli S Seagraves DA Hastings TA Chen XH Garlan RW Psychosocial predictors of resilience after the September 11, 2001 terrorist attacks J Nerv Mental Dis 2009 197 266 273 10.1097/NMD.0b013e31819d9334 19363383

[B36] Updegraff JA Silver RC Holman EA Searching for and finding meaning in collective trauma: Results from a national longitudinal study of the 9/11 terrorist attacks J Pers Soc Psychol 2008 95 709 722 18729704 10.1037/0022-3514.95.3.709 PMC2617710

[B37] Buck N Kindt M Arntz A van den Hout M Schouten E Psychometric properties of the Trauma Relevant Assumptions Scale J Anxiety Disord 2008 22 1496 1509 10.1016/j.janxdis.2008.03.010 18424063

[B38] Horowitz MJ Stress response syndromes. Personality styles and interventions 2001 4 Jason Aronson Inc, Northvale, New Jersey

[B39] NGDC Natural hazards data, images and education Book Natural hazards data, images and education 2010 National Geophysical Data Center Web site, City

[B40] Heir T Weisæth L Acute disaster exposure and mental health complaints of Norwegian tsunami survivors six months post disaster Psychiatry 2008 71 266 276 10.1521/psyc.2008.71.3.266 18834277

[B41] Heir T Piatigorsky A Weisæth L Longitudinal changes in recalled perceived life threat after a natural disaster Bri J Psychiatry 2009 194 510 514 10.1192/bjp.bp.108.056580 19478289

[B42] Heir T Sandvik L Weisæth L Hallmarks of posttraumatic stress: Symptom Z-scores in a tsunami-affected tourist population Psychopathology 2009 42 157 164 10.1159/000207457 19276641

[B43] Hussain A Weisaeth L Heir T Nonresponse to a population-based postdisaster postal questionnaire study J Traum Stress 2009 22 324 328 10.1002/jts.20431 19644976

[B44] Weisæth L The stressor-response relationship Post-traumatic stress disorder 1996 Bailliere Tindal, London 191 216

[B45] Heir T Rosendal S Bergh-Johannesson K Michel PO Mortensen EL Weisæth L Andersen HS Hultman CM Tsunami-affected Scandinavian tourists: Disaster exposure and post-traumatic stress symptoms Nordic J Psychiatry 2011 65 9 15 10.3109/08039481003786394 20429748

[B46] Cantril H The pattern of human concerns 1965 Rutgers University Press, New Brunswick, NJ

[B47] de Boer AG van Lanschot JJ Stalmeier PF van Sandick JW Hulscher JB de Haes JC Sprangers MA Is a single-item visual analogue scale as valid, reliable and responsive as multi-item scales in measuring quality of life? Qual Life Res 2004 13 311 320 15085903 10.1023/B:QURE.0000018499.64574.1f

[B48] Weiss DS Marmar CR Wilson JP, Keane TM The Impact of Event Scale-Revised Assessing psychological trauma and PTSD 1997 Guilford Press, New York, NY 399 411

[B49] Weiss DS Wilson JP, Keane TM The Impact of Event Scale-Revised Assessing psychological trauma and PTSD 2004 The Guilford Press, New York, NY 168 189

[B50] Eid J Larsson G Johnsen BH Laberg JC Bartone PT Carlstedt B Psychometric properties of the Norwegian Impact of Event Scale-Revised in a non-clinical sample Nordic J Psyc 2009 63 426 432 10.1080/08039480903118190 19688636

[B51] Schafer JL Analysis of incomplete multivariate data 1997 Chapman & Hall, London, UK

[B52] Nygaard E Posttraumatic stress reactions of Norwegian children and families after the Southeast Asian tsunami 2012 University of Oslo, Department of Psychology

[B53] Wahl AK Rustoen T Rokne B Lerdal A Knudsen O Miaskowski C Moum T The complexity of the relationship between chronic pain and quality of life: A study of the general Norwegian population Qual Life Res 2009 18 971 980 10.1007/s11136-009-9515-x 19688608 PMC2744798

[B54] Ceyhan E Ceyhan AA Earthquake survivors' quality of life and academic achievement six years after the earthquakes in Marmara, Turkey Disasters 2007 31 516 529 10.1111/j.1467-7717.2007.01023.x 18028168

[B55] Janoff-Bulman R Posttraumatic growth: Three explanatory models Psychological Inquiry 2004 15 30 34

[B56] Helgeson VS Reynolds KA Tomich PL A meta-analytic review of benefit finding and growth J Consult Clin Psychol 2006 74 797 816 17032085 10.1037/0022-006X.74.5.797

[B57] Gudmundsdottir B Beck JG Coffey SF Miller L Palyo SA Quality of life and post trauma symptomatology in motor vehicle accident survivors: The mediating effects of depression and anxiety Depress Anxiety 2004 20 187 189 10.1002/da.20037 15580574

